# Analysis of the swine tracheobronchial lymph node transcriptomic response to infection with a Chinese highly pathogenic strain of porcine reproductive and respiratory syndrome virus

**DOI:** 10.1186/1746-6148-8-208

**Published:** 2012-10-30

**Authors:** Laura C Miller, Damarius Fleming, Andrew Arbogast, Darrell O Bayles, Baoqing Guo, Kelly M Lager, Jamie N Henningson, Sarah N Schlink, Han-Chun Yang, Kay S Faaberg, Marcus E Kehrli

**Affiliations:** 1 Virus and Prion Research Unit, National Animal Disease Center-USDA-ARS, Ames, Iowa, 50010, USA; 2 Inter-departmental Genetics, Iowa State University, Ames, Iowa, 50011, USA; 3 Department of Computer Science, Iowa State University, Ames, Iowa, 50011, USA; 4 Infectious Bacterial Diseases Research Unit, National Animal Disease Center-USDA-ARS, Ames, Iowa, 50010, USA; 5 Veterinary Diagnostic & Production Animal Medicine, Iowa State University, Ames, Iowa, 50011, USA; 6 China Agricultural University, Beijing, China

## Abstract

**Background:**

Porcine reproductive and respiratory syndrome virus (PRRSV) is a major pathogen of swine worldwide. Emergence in 2006 of a novel highly pathogenic PRRSV (HP-PRRSV) isolate in China necessitated a comparative investigation into the host transcriptome response in tracheobronchial lymph nodes (TBLN) 13 days post-infection with HP-PRRSV rJXwn06, PRRSV strain VR-2332 or sham inocula. RNA from each was prepared for next-generation sequencing. Amplified library constructs were directly sequenced and a list of sequence transcripts and counts was generated using an RNAseq analysis pipeline to determine differential gene expression. Transcripts were annotated and relative abundance was calculated based upon the number of times a given transcript was represented in the library.

**Results:**

Major changes in transcript abundance occurred in response to infection with either PRRSV strain, each with over 630 differentially expressed transcripts. The largest increase in transcript level for either virus versus sham-inoculated controls were three serum amyloid A2 acute-phase isoforms. However, the degree of up or down-regulation of transcripts following infection with HP-PRRSV rJXwn06 was greater than transcript changes observed with US PRRSV VR-2332. Also, of 632 significantly altered transcripts within the HP-PRRSV rJXwn06 library 55 were up-regulated and 69 were down-regulated more than 3-fold, whilst in the US PRRSV VR-2332 library only 4 transcripts were up-regulated and 116 were down-regulated more than 3-fold.

**Conclusions:**

The magnitude of differentially expressed gene profiles detected in HP-PRRSV rJXwn06 infected pigs as compared to VR-2332 infected pigs was consistent with the increased pathogenicity of the HP-PRRSV in vivo.

## Background

Porcine reproductive and respiratory syndrome virus (PRRSV), the causative agent of PRRS in swine, is a member of the *Arteriviridae* family in the order *Nidovirales*. PRRSV causes highly significant economic losses to the swine industry worldwide [1] as a result of both reproductive failure (late-term abortions and stillbirths) in pregnant sows and respiratory disease (pneumonia) in nursery and grower/finishing pigs [2]. Infection with PRRSV also predisposes pigs to infection by bacterial pathogens as well as other viral pathogens [3-7], as such, PRRSV is a key etiological agent of the porcine respiratory disease complex (PRDC). Clinical disease caused by PRRSV is highly variable, ranging from mild, subclinical infection to acute death of adult animals [8]. Differences in virulence have been attributed to numerous factors including host genetics, management practices, and virus strain heterogeneity [9-16]. Relatively little is known about the interactions of PRRSV and host cells. The lymph node is an anatomic site where the innate immune response and adaptive immune system interface. Tracheobronchial lymph nodes (TBLN) in swine drain the lung field and provide the focal structure that can reproducibly be identified. Although the TBLN contains a number of cell types, sampling this tissue allows study of direct and indirect effects of an infectious agent on the lung and cells within the lymph node.

In 2006 a unique syndrome with high morbidity and mortality was recognized in growing pigs in China that was originally known as porcine high fever disease (PHFD) due to its uncertain etiology [17]. Experimental infection of pigs in China with these novel viral isolates reproduced the clinical disease providing strong evidence for the role of PRRSV as the causal agent of PHFD. However, there was still a question as to whether there was some unknown agent in the PRRSV preparations that increased the severity of the clinical disease over what was expected for a “routine” PRRSV infection. This question was resolved when PHFD was reproduced in China with virus derived from an infectious clone of the JX143 PRRSV isolate [18] demonstrating that PRRSV isolates with a common genetic motif had a causal role in PHFD leading to this lineage of virus being called highly pathogenic PRRSV (HP-PRRSV). We imported a plasmid containing a full-length clone of the 2006 JXwn06 HP-PRRSV isolate [19] from which infectious virus (rJXwn06) was rescued. An animal study was conducted comparing the pathogenicity of HP-PRRSV isolate rJXwn06 with the North American prototype strain VR-2332 PRRSV [20]. The objective of this report was to investigate gene expression profiles in porcine tracheobronchial lymph node (TBLN) during viral infection with HP-PRRSV rJXwn06 strain alongside of US PRRSV strain VR-2332 at a snapshot of 13 days post-infection using bioinformatics.

## Results and discussion

### Mapping short RNA-seq reads and estimating transcript expression levels

Genomic Short-read Nucleotide Alignment Program (GSNAP) was used for alignment and genome construction, and Cufflinks to determine if differential expression and changes in transcript abundance were statistically significant [21,22]. The RNASeq yielded 55,527,464 reads for the control, 43,263,207 reads for the HP-PRRSV, and 34,555,783 for VR-2332 libraries after quality trimming and excluding any reads less than 25 bp. Cufflinks was used to measure transcript abundances in fragments per kilobase of exon per million fragments mapped (FPKM). The Cuffdiff output contained normalized FPKM for comparison between libraries (Additional file 1). These values were used to calculate the fold change (log2 transformed) in expression between the experimental unit and the control.

Examination of the RNASeq data indicated that there were major changes in transcript abundance occurring in the PRRSV-infected TBLN-based unique transcripts [Cuffdiff output (Additional file 1)]. Of these total transcripts, 632 were found to be significant hits in the HP-PRRSV rJXwn06 library and 633 were significant in the US PRRSV VR-2332 library (Table 1). Of those 632 significant hits within the HP-PRRSV rJXwn06 library 55 hits were up-regulated and 69 were down-regulated more than 3-fold whilst in the US PRRSV VR-2332 library 4 hits were up-regulated and 116 were down-regulated more than 3-fold.

**Table 1:** Table of transcript counts

** Sample**	**Significant Hits**	**Up-regulated genes >3-fold**	**Down-regulated genes >3-fold**
HP-PRRSV rJXwn06 vs. Control	632	55	69
US PRRSV VR-2332 vs. Control	633	4	116

This derived catalog of expressed genes represents the first comparative analysis of the HP-PRRSV rJXwn06 and VR-2332-infected TBLN transcript abundance profiles and provides a database that informs us of genes involved in normal TBLN physiology, as well as genes whose abundance is altered by PRRSV infection.

### Annotation of significantly differentially expressed genes

Gene annotation of all significant hits (Additional files 1 and 2) was then carried out using a MySQL database matching the Ensmbl (Sscrofa9.56) chromosome location of aligned transcripts to gene names. Gene IDs and log2 fold-change expression values for significant hits, that had FPKM values in both the control and the infected differential expression testing for transcripts (Cuffdiff output files), were then analyzed using the Ingenuity Pathway Analysis software. When comparing the TBLN transcriptome from sham-inoculated controls vs. the HP-PRRSV rJXwn06-infected pigs, 568 of the 632 gene IDs mapped to the Ingenuity Knowledge Base and 165 were up-regulated while 148 were down-regulated. In the TBLN of control vs. VR-2332-infected pigs, 528 of the 633 gene IDs mapped to the Ingenuity Knowledge Base and only 8 were up-regulated while 235 were down-regulated.

Table 2 lists the top ten genes (named by the HUGO Gene Nomenclature Committee (HGNC) [23]) we detected that had a significant value in both HP-PRRSV rJXwn06 and VR-2332-infected TBLN RNAseq Cuffdiff output and a fold-change increase or decrease of greater than 3. Transcripts up-regulated in both HP-PRRSV rJXwn06 and VR-2332-infected TBLN by > 9-fold and > 4-fold vs. control TBLN, respectively, were three serum amyloid A2 (SAA4) acute-phase isoforms, as well as gene ENSSSCG00000013369 F1S9C0_PIG serum amyloid protein (no HGNC annotation), that are expressed in response to inflammatory stimuli (Table 2). Other annotated genes (Table 2) that were up-regulated in HP-PRRSV rJXwn06 TBLN vs. control were resistin (RETN) which is secreted by immune and epithelial cells and participates in the immune response by increasing transcriptional events that increase expression of several pro-inflammatory cytokines [24]; three members of the S100 family (S100A9, S100A8, S100A12) of calcium-binding proteins localized in the cytoplasm and/or nucleus of a wide range of cells, involved in the regulation of a number of cellular processes such as cell cycle progression and differentiation, and mediators of inflammatory and protective anti-infection responses [25]; xanthine dehydrogenase (XDH) a generator of reactive oxygen species and possible cause of hypoxia-mediated lung injury [26]; and peptidylarginine deiminase, type IV, (PADI4) which may play a role in granulocyte and macrophage development leading to inflammation and immune responses [27]. Also in the top ten up-regulated transcripts were two genes without a HGNC symbol, TREM1_PIG (ENSSSCG00000001617) trigger receptor, which is expressed on myeliod cells, and the interleukin-1 receptor, type II gene (IL1R2) which is associated with host responses to subdue inflammation as a consequence of disease. Down-regulated in HP-PRRSV TBLN vs. control TBLN were diacylglycerol O-acyltransferase 2 (DGAT2) which catalyzes triglyceride synthesis which is critical for formation of adipose tissue [28]; perilipin-1 (PLIN1) an important regulator of lipid storage; a member of the cytochrome P450 monooxygenases (CYP4B1) of unknown specific function; soluble galactose-binding lectin 12 (LGALS12), cell death-inducing DFFA-like effector C (CIDEC), tumor suppressor candidate 5 (TUSC5), protein phosphatase 1, regulatory (inhibitor) subunit 1A (PPP1R1A), C-type lectin domain family 4, member G, that encodes a glycan-binding receptor and a member of the C-type lectin family which plays a role in T-cell immune responses (CLEC4G). Also in the top ten down-regulated transcripts were the following genes without projected HGNC symbols: CES1 liver carboxylesterase (ENSSSCG00000002825) and F1STY2_PIG thyroid hormone-responsive protein (ENSSSCG00000014888). In VR-2332-infected pig TBLN vs. control TBLN, transcript abundance was down-regulated to a lesser extent and featured genes linked to metabolism in adipose tissue and regulation in neuronal activity functions including dermatopontin (DPT) extracellular matrix protein with possible functions in cell-matrix interactions and matrix assembly which enhances transforming growth factor beta (TGFB1) activity; beta-1 adrenergic receptor (ADRB1); Solute carrier family 2, facilitated glucose transporter member 4 (SLC2A4); uncharacterized MLX interacting protein-like protein (MLXIPL); basic helix-loop-helix transcription factor 15 (TCF15); forkhead box transcription factor protein C2 (FOXC2); protein phosphatase 1 regulatory subunit 1B also known as dopamine- and cAMP-regulated neuronal phosphoprotein (PPP1R1B); potassium voltage-gated channel, KQT-like subfamily, member 4 (KCNQ4) that is thought to play a critical role in the regulation of neuronal excitability; plexin domain containing 1 (PLXDC1); and adenosine A1 receptor (ADORA1).

**Table 2:** Top Ten HGNC named genes with their fold change obtained from the RNAseq data

** HP-PRRSV rJXwn06**		** US PRRSV VR-2332**	
**Named gene**	**Fold change (log2)**	**Named gene**	**Fold change (log2)**
SAA4	↑9.07171	SAA4	↑4.15255
RETN	↑6.89887		
S100A9	↑6.41344		
XDH	↑5.97329		
S100A8	↑5.75953		
ENSSSCG00000013369	↑5.72684		
S100A12	↑5.37987		
IL1R2	↑4.571		
PADI4	↑4.48918		
ENSSSCG00000001617	↑4.43141		
DGAT2	↓-4.79123	DPT	↓-3.03886
PLIN1	↓-4.84105	ADRB1	↓-3.04682
CYP4B1	↓-5.07721	SLC2A4	↓-3.06235
ENSSSCG00000002825	↓-5.11753	MLXIPL	↓-3.07544
LGALS12	↓-5.31501	TCF15	↓-3.08758
CIDEC	↓-5.43776	FOXC2	↓-3.10329
TUSC5	↓-5.52995	PPP1R1B	↓-3.10611
PPP1R1A	↓-5.64244	KCNQ4	↓-3.12634
CLEC4G	↓-7.21094	PLXDC1	↓-3.14039
ENSSSCG00000014888	↓-7.33358	ADORA1	↓-3.1413

**Figure 1:**
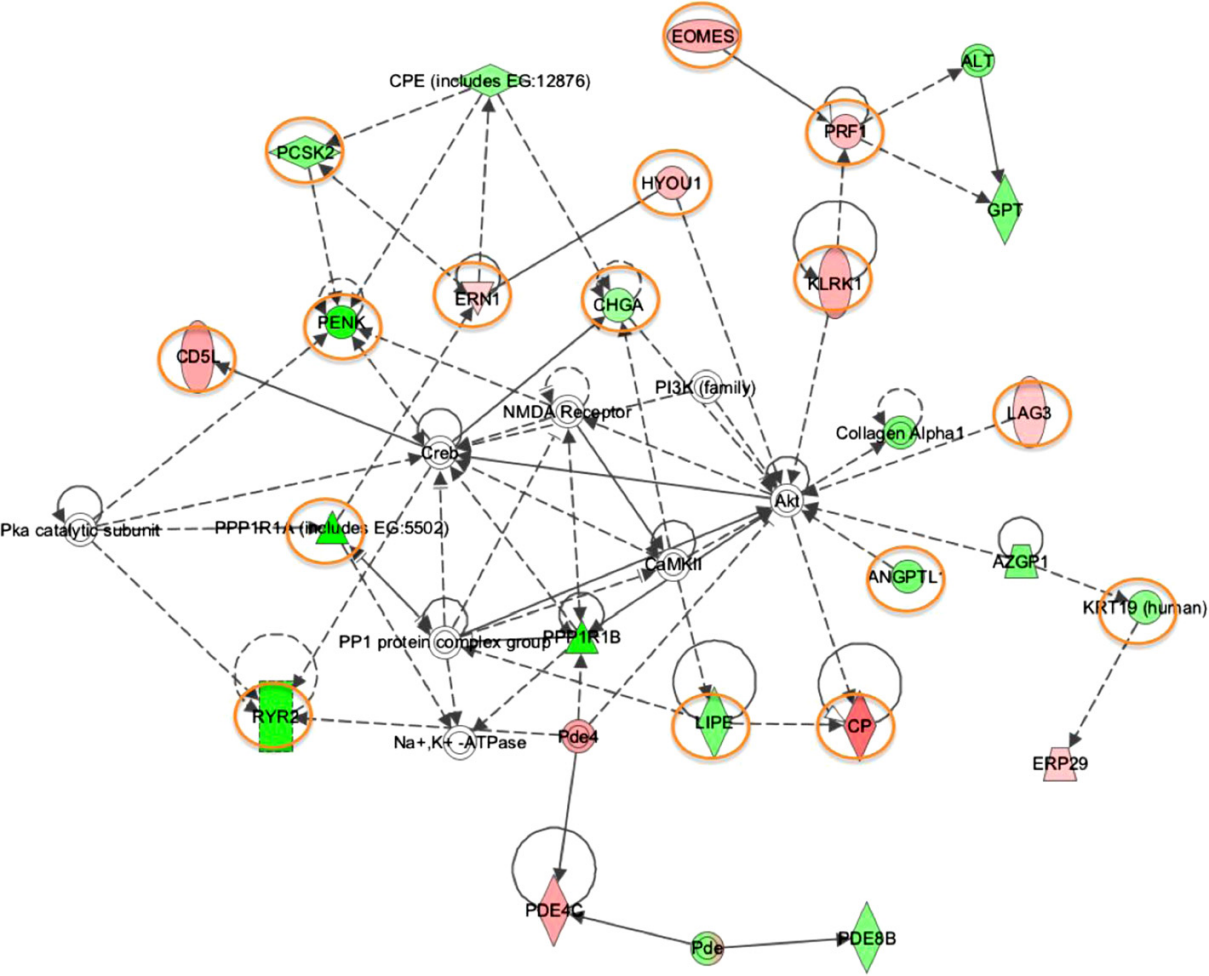
**Ingenuity pathways analysis summary.** To investigate possible interactions of differently regulated genes, datasets representing 568 genes with altered expression profile obtained from the RNAseq data for HP-PRRSV rJXwn06 were imported into the Ingenuity Pathway Analysis Tool and the following data is illustrated: The network representation of the most highly rated network (Gene Expression, Cell Death, Lipid Metabolism). The genes that are shaded were determined to be significant from the statistical analysis. The genes shaded red are up-regulated and those that are green are down-regulated. The intensity of the shading shows to what degree each gene was up or down-regulated. A solid line represents a direct interaction between the two gene products and a dotted line means there is an indirect interaction. Genes associated with Cell Death are circled with orange color.

Analysis of the genomic data in the context of gene ontology, by Ingenuity Pathway Analysis (IPA), allowed us to ascribe biological functional networks to the differentiated transcript abundance dataset. The top functions identified with the Ingenuity Canonical Pathway list, filtered to apoptosis, cellular immune response, cytokine signalling, humoral immune responses and pathogen-influenced signalling, based on differentially expressed genes were: granzyme A signalling, crosstalk between dendritic cells and natural killer cells, IL-10 signalling, role of pattern recognition receptors in recognition of bacteria and viruses, IL-12 signalling and production in macrophages, complement system, interferon signalling, communication between innate and adaptive immune cells, IL-17A signalling in fibroblasts, granzyme B signalling, production of nitric oxide and reactive oxygen species in macrophages, differential regulation of cytokine production in macrophages and T helper cells by IL-17A and IL-17F that were above the threshold of p value < 0.05, as calculated by Fischer's test representing the ratio of number of genes from the dataset that map to the pathway and the number of all known genes ascribed to the pathway. The genes up-regulated in the HP-PRRSV rJXwn06 infected pigs’ TBLN were associated in 18 networks: from biological networks with functions associated with cell death, antimicrobial responses and cancer, with the highest network score of 37, i.e. the likelihood of genes in this network would have approximately a 10^-37^ chance of occurring randomly, and 21 focus molecules, i.e. the starting points for generating biological networks; to networks with functions associated with nervous system development and function, organ morphology and reproductive system disease with a score of 2 and 1 focus molecule. Many of the up-regulated networks related to cell death and inflammatory response functions fit with the results previously reported [17,29] where HP-PRRSV strain rJXwn06 caused severe disease, resulted in up to 100X higher abundance of virus and produced an exacerbated release of cytokines, including pro-inflammatory cytokines, when compared to Type 2 prototype strain VR-2332. Wide spread tissue damage [30] and cell death were observed as predicted by up-regulation of cell-death associated genes (circled in orange in Figure 1) in the network representation of the mostly highly rated network for HP-PRRSV rJXwn06 by IPA. The down-regulated network functions in the HP-PRRSV rJXwn06 infected TBLN included activities associated with cellular function and maintenance, tissue morphology, metabolic disease, organismal development, carbohydrate metabolism, lipid metabolism, small molecule biochemistry, post-translational modification, protein folding, developmental disorder, which may be associated with cell death and reflects a severe disease state. Similarly, the down-regulated network functions in the VR-2332 infected TBLN were associated with cellular function, maintenance, development and organization.

This study produced transcriptional profiles of TBLNs from non-infected, HP-PRRSV rJXwn06 and US PRRSV VR-2332-infected pigs that provides insight into immune dysregulation elicited by the virus on host transcript abundance levels necessary for a effective immune response.

## Conclusions

This RNA-Seq compendium extends the analyses of previous gene expression atlases performed using Affymetrix GeneChip technology and provides an example of new methods to accommodate the increase in transcriptome data obtained from next generation sequencing [29,31,32].

It is well established that many pathogens cause changes in expression of specific genes that act to protect the host and clear the infection. PRRSV strains differ in their dysregulation of the immune response to infection and delay in development of a protective immune response in vaccinated pigs [33,34]. A higher number of significantly differentially expressed gene instances were detected in HP-PRRSV rJXwn06 than VR-2332 when normalized to control samples at a snapshot of 13 days post inoculation (dpi). As anticipated, some of the genes (e.g., resistin) and pathways identified would be expected to be involved in the host response to a severe disease. In the case of resistin, it would be expected that adipose tissue stores are being mobilized as part of the host response to infection, which includes a high fever typical of infection with HP-PRRSV. There are specific cellular proteins that regulate a protective immune response, for example the pro-inflammatory genes that were up-regulated to a greater extent in HP-PRRSV rJXwn06 than VR-2332 when normalized to control samples as observed when comparing the pathogenicity of HP-PRRSV isolate rJXwn06 with the North American prototype strain VR-2332 PRRSV. At 13 dpi HP-PRRSV rJXwn06 inoculated pigs had an interstitial pneumonia that was significantly more severe than theVR-2332 inoculated group which appeared to be convalescing [30]. Future studies of these differentially expressed genes, their transcript abundance, protein level, and protein function will enhance our understanding of the interaction of PRRSV with the host. Identification of new virulence mechanisms of PRRSV may improve the prospects for rational design of more effective vaccines to limit viral replication and shedding.

## Methods

### Cells and virus

MARC-145 cells were cultured in minimum essential medium (EMEM, SAFC 56416C) with 10% fetal bovine serum at 37°C, 5% CO_2_. Wild-type (*wt*) Type 2 PRRSV strain VR-2332 (GenBank U87392), passage 6 on MARC-145 cells, was titrated and used for the swine study. Virus (rescued JXwn06; rJXwn06) was rescued from a cloned cDNA of Chinese highly pathogenic Type 2 PRRSV strain JXwn06 [pWSK-JXwn; GenBank EF641008, [19]] and passaged 3 times on MARC-145 cells for use in the swine study.

### Swine study

The animal use protocol was reviewed and approved by the Institutional Animal Care and Use Committee (IACUC) of the National Animal Disease Center-USDA-Agricultural Research Service. Thirty-two 10-week-old cross-bred pigs were obtained from a U.S. high-health herd and were found to be free of PRRSV and influenza virus antibodies using commercially available enzyme-linked immunosorbent assay (ELISA) kits (HerdChek PRRS 2XR; IDEXX Laboratories, Westbrook, Maine) and NP ELISA (MultiS ELISA, IDEXX, Westbrook, Maine), respectively. Pigs were also confirmed negative for porcine circovirus type 2 by quantitative real-time PCR [35]. One day prior to starting the experiment, pigs were bled, weighed and randomly assigned to one of four groups. Group 1 (N = 8) consisted of negative control pigs, which received an intranasal 2 ml sham inoculum of minimum essential media (MEM) on 0 dpi. Group 2 pigs (N = 12) were challenged intranasally with 2 ml of 1 × 10^6^ 50% tissue culture infective dose (TCID_50_)/ml of Chinese PRRSV strain rJXwn06 in Animal Biosafety Level-3-Agriculture (ABSL-3-Ag) housing, where they remained for the duration of the experiment. Group 3 consisted of naïve pigs (N = 4) that were placed in contact with Group 2 swine on 2 dpi. Group 4 pigs (N = 8) were challenged intranasally with 2 ml of 1 × 10^6^ TCID_50_/ml of Type 2 prototype strain VR-2332. Groups 1 and 2 were housed in separate isolation rooms in an ABSL2 facility. Animal care and euthanasia were conducted in accordance with the Report of the AVMA Panel on Euthansia and under the supervision of IACUC of NADC. Serum and bronchoalveolar lung lavage fluid (BALF) were tested for infectious virus as described previously [36]. Lungs were scored for gross lesions [11] and sections fixed for histopathology. Swabs were collected from BALF, and various sites for bacterial isolation [37].

### RNA isolation

Following humane euthanasia, tracheobronchial lymph nodes (TBLN) from in vivo HP-PRRSV rJXwn06 (N = 8), US PRRSV VR-2332 (N = 7), or sham-infected pigs (N = 8) were harvested at 13 days post-infection and total cellular RNA was prepared as follows. One gram of TBLN from each pig was collected immediately upon necropsy, minced and stored in RNAlater (Life Technologies, Grand Island, NY) at −80°C until homogenized for extraction of total RNA with MagMAX™-96 for Microarrays Total RNA Isolation Kit (Applied Biosystems, Carlsbad, CA) using the manufacturer's protocol. The integrity of the RNA was confirmed with a 2100 Bioanalyzer and RNA 6000 Nano-chip (Agilent, Santa Clara, CA). The samples used had an average RNA Integrity Number (RIN) value of 7.8 and 28S:18S rRNA ratio of 1.9.

### cDNA library construction

cDNA libraries were constructed from pooled total cellular RNA from the TBLN in each treatment group using TruSeq Sample Prep Kits (Illumina Inc., San Diego, CA) and sequenced by 2 × 100 paired-end sequencing on an Illumina HiSeq 2000 instrument.

### RNA-Seq (Whole Transcriptome Shotgun Sequencing) pipeline

**Figure 2:**
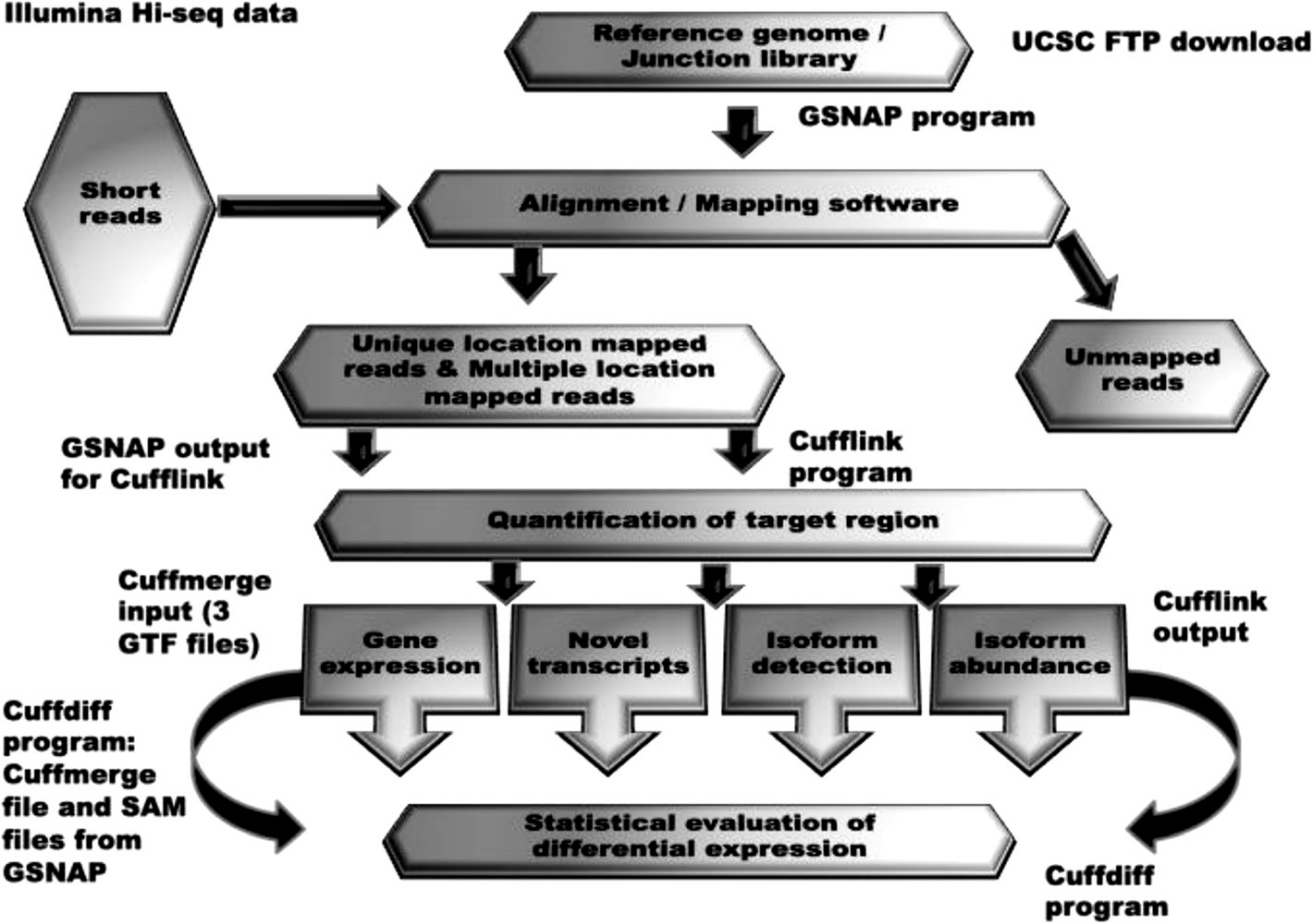
**Computational pipeline.** RNAseq analysis pipeline comprised of GSNAP for alignment and Cufflinks to determine if differential expression, and changes in transcript abundance were statistically significant (adapted from [38]).

In order to analyze the Illumina reads, a series of bioinformatics methods were used to investigate gene expression profiles in TBLN during PRRSV infection with HP-PRRSV rJXwn06 and US PRRSV VR-2332 at a snapshot of 13 dpi. This was carried out with the construction of a RNAseq analysis pipeline (Figure 2) comprised of GSNAP for alignment and genome construction, and Cufflinks to determine if differential expression and changes in transcript abundance were statistically significant. Three files of transcriptome data from the sham, HP-PRRSV rJXwn06 and US PRRSV VR-2332 inoculated groups were aligned to the UCSC pig genome build using the GSNAP alignment program in preparation for differential expression analysis. The next step in the pipeline was to put the GSNAP output into the Cufflinks program and run it through three separate utilities or tools within the software package; Cufflinks, Cuffmerge, and Cuffdiff. First the three files were run through Cufflinks in order to assemble the aligned RNA Sequence reads into transcripts and estimate the abundances in FPKM of the paired–end reads. The Cufflinks q-value was the false discovery rate (FDR)-adjusted p-value of the uncorrected test statistic. The q-value used in this study was 0.05. The significance status was “yes” when p was greater than q after Benjamini-Hochberg correction for multiple-testing (Additional file 1). Cuffmerge was then used to create a single transcript dataset from the multiple reconstructions. Two runs were then conducted using the HP-PRRSV rJXwn06 vs. control and the US PRRSV VR-2332 vs. control datasets using the Cuffdiff program to test for differential expression and regulation amongst the two disease states. Gene annotation of all significant hits was then carried out using a MySQL database matching to the Ensembl Sscrofa 9.56 reference genome currently supported by the Integrative Genomics Viewer (Broad Institute).

### Ingenuity pathway analysis (IPA)

Datasets representing genes with altered expression profile derived from RNAseq analyses were imported into the Ingenuity Pathway Analysis Tool (IPA Tool; Ingenuity® Systems, Redwood City, CA, USA; http://www.ingenuity.com). In IPA, differentially expressed genes were mapped to genetic networks available in the Ingenuity database and then ranked by score.

The basis of the IPA program consists of the Ingenuity Pathway Knowledge Base (IPKB) that is derived from known functions and interactions of genes published in the literature. Thus, the IPA Tool allows the identification of biological networks, global functions within the host and functional pathways of a particular dataset. The program also gives the significance value of the differentially expressed genes, the other genes with which it interacts, and how the products of the genes directly or indirectly act on each other, including those not involved in the microarray analysis. The networks created are ranked depending on the number of significantly expressed genes they contain and also list diseases that were most significant (Figure 1).

## Competing interests

The authors declare that they have no competing interests.

## Authors' contributions

LCM: study conception, data collection and analysis, research design, manuscript writing. DF: research design, data analysis. AA: data analysis. DOB: RNAseq interpretation discussions and data analysis. BG: infectious clone and production of virus stocks. KML: study conception, animal study execution, virus stocks, data collection, and manuscript preparation and writing. JNH: data collection. SNS: data collection. H-CY: infectious clone of HP-PRRSV strain JXwn06. KSF: study conception, rescue of HP-PRRSV infectious clone, and production of virus stocks. MEK: study conception and manuscript preparation and writing. All authors read and approved the final manuscript.

## Supplementary Material

Additional file 1 **Cuffdiff output of all significant hits for comparison, using the HP-PRRSV rJXwn06 vs. control and the US PRRSV VR-2332 vs. control datasets, to test for differential expression and regulation amongst the two disease states.** Gene annotation (symbol) was carried out using a MySQL database matching to the Ensembl Sscrofa9.56 reference genome currently supported by the Integrative Genomics Viewer (Broad Institute). Location (row.names); gene annotation (symbol); entrez gene name; treatment (sample_1, sample_2); status; abundance in FPKM (value_1, value_2); differential expression (log2_fold_change); test statistic (test_stat); p-value (p_value); false discovery rate (FDR)-adjusted p-value of the uncorrected test statistic (q_value); significance status after Benjamini-Hochberg correction for multiple-testing (significant). Click here for file

Additional file 2 Transcript sequences of all significant hits inthe HP-PRRSV rJXwn06 vs. control and the US PRRSV VR-2332 vs. control datasets. Click here for file
